# Effectiveness of Online-Delivered Project ImPACT for Children With ASD and Their Parents: A Pilot Study During the COVID-19 Pandemic

**DOI:** 10.3389/fpsyt.2022.806149

**Published:** 2022-03-24

**Authors:** Fēi Li, Danping Wu, Fang Ren, Lixiao Shen, Minbo Xue, Juehua Yu, Lingli Zhang, Yun Tang, Xin Liu, Minyi Tao, Li Zhou, Liping Jiang, Mingyu Xu, Fei Li

**Affiliations:** ^1^School of Nursing, Shanghai Jiao Tong University School of Medicine, Shanghai, China; ^2^Department of Developmental and Behavioral Pediatric and Child Primary Care, Brain and Behavioral Research Unit of Shanghai Institute for Pediatric Research, Ministry of Education (MOE)-Shanghai Key Laboratory for Children's Environmental Health, Xinhua Hospital Affiliated to Shanghai Jiao Tong University School of Medicine, Shanghai, China; ^3^Faculty of Education, Yunnan Normal University, Kunming, China; ^4^Psychology and Neuroscience of Cognition Research Unit, University of Liège, Liège, Belgium; ^5^Department of Nursing, Xinhua Hospital Affiliated to Shanghai Jiao Tong University School of Medicine, Shanghai, China

**Keywords:** autism spectrum disorder, parent-mediated intervention, e-health, social communication, parenting stress

## Abstract

**Objective:**

During the COVID-19 pandemic, face-to-face intervention services for families of children with autism spectrum disorder (ASD) were limited. This study aimed to evaluate the effectiveness of an 8-week, online-delivered Project ImPACT program for children with ASD and their parents in China during the COVID-19 pandemic.

**Methods:**

A pilot non-randomized study with a waitlist control group was conducted in 68 children with ASD and their parents in the Department of Developmental and Behavioral Pediatrics between April 15, 2020 and March 19, 2021. Participants were allocated to either the intervention (IG) or the waitlist group (WLG) according to their order of recruitment. Parents in the IG immediately received 8 weeks of the online-delivered Project ImPACT program, and the WLG received the same program with a delay when the IG had completed all sessions. Participants in both groups received treatment as usual during the research period.

**Results:**

The online-delivered Project ImPACT program significantly improved the parent-reported social communication skills of children with ASD. Furthermore, parent's involvement in the training program produced a collateral reduction in parenting stress and an increase in perceived competence in the parental role. Parents rated the program acceptable in terms of curriculum schedule, session content, homework assignments, and therapist feedback.

**Conclusions:**

The 8-week, online-delivered Project ImPACT program is a feasible and effective social skill training program for families of children with ASD in China during the COVID-19 pandemic. Due to the methodological limitations, randomized controlled studies with larger sample sizes are suggested to provide more solid evidence.

## Introduction

Autism spectrum disorder (ASD) is a lifelong neurodevelopmental disability that is characterized by impairments in social communication and interaction as well as the presence of restricted interests and stereotyped behaviors (1). Currently, the prevalence of ASD is estimated to be 18.5 per 1,000 among children aged 8 years (2). Deficits in social communication and interaction are the most commonly reported red flags and significant core symptoms of ASD (1). These core impairments not only disrupt children's ability to develop and maintain social relationships but also impact their long-term outcomes (3, 4). Moreover, the difficulties and challenges that accompany the child's social impairment can lead to considerable parenting stress and caregiving burden, which further disrupts parental psychological wellbeing and quality of life (5). Therefore, it is crucial for early intervention services to target social skill training for children with ASD.

Best practice recommends at least 25 h per week of comprehensive interventions for children with ASD (6), but many families fail to meet this standard due to the shortage of quality services and well-trained professionals (7, 8) in China, especially some rural region. Parent-mediated intervention (PMI) is a cost-effective model to fill the existing gap. It is widely believed that PMIs can enhance many aspects of children's social development, such as social communication, adaptive behaviors, cognition, and language (9, 10). Furthermore, the involvement of parents contributes to the increase in parental self-efficacy and reduction in parenting stress (11, 12). Traditional PMIs are delivered on a face-to-face basis and parents have to travel a long distance to clinical centers, which is restricted by limited medical resources, considerable time consumption, and geographical barriers (13). Furthermore, these restrictions have become even more pressing during the COVID-19 pandemic because face-to-face intervention services for children with ASD were interrupted in most areas of China. Telehealth is a promising model of service delivery, which allows medical care services to be delivered remotely and efficiently (14). There are many advantages to remote interventions, including low cost, high reach of the target population, diverse teaching resources, and standardized instructions with high fidelity (13). Existing evidence suggests that telehealth intervention programs are highly acceptable, comparable to face-to-face interventions, and effective for the outcomes of both the implementer and the child (15). Telehealth has been successfully implemented in the care of many other chronic diseases such as dementia care (16) and psychotherapy (17). In the early assessment, diagnosis and intervention for children with ASD, telemedicine also showed satisfactory effects (18, 19).

Among the various PMI programs, Project Improve Parents as Communication Teacher (ImPACT) is an evidence-based, parent-mediated, social skill training program developed by Brooke Ingersoll and Anna Dvortcsak (20, 21). It is designed for young children with ASD and helps parents to encourage the development of children's social communication skills (e.g., social engagement, communication, imitation, and play) during daily activities and routines (20, 21). The initial feasibility and efficacy of the Project ImPACT program have been examined in previous studies (22–26). Specifically, improvements in the spontaneous use of language targets and social communication skills were observed in children with ASD (22, 24). In addition, parents exhibited a decrease in parenting stress (23), an increase in parental self-efficacy, and a positive perception toward their children (26). The feasibility, acceptability, and usability of the program were highly favorable among the target population (23).

Despite promising evidence for the effectiveness of the Project ImPACT program, most studies were conducted in developed countries. The small sample sizes and lack of positive control groups limit the generalizability of the conclusions. Furthermore, it remains to be studied whether the Project ImPACT program delivered remotely also positively affects autistic children and their parents. To enrich early intervention services and address new challenges of the global health crisis, we adapted the Project ImPACT program and delivered it remotely. This pilot waitlist control study was a preliminary examination of the effectiveness of an 8-week, online-delivered Project ImPACT program for families of children with ASD in China during the COVID-19 Pandemic. We hypothesized that the online-delivered Project ImPACT program will reduce the severity of social impairment for children with ASD, improve parental self-efficacy, and reduce parenting stress. Therefore, the primary aim of this study was to identify the effect of the online-delivered Project ImPACT program on children's social communication skills, and the secondary aim was to explore whether the online-delivered Project ImPACT program increases parental self-efficacy and reduces parenting stress.

## Methods

### Study Design and Setting

A pilot non-randomized study with a waitlist control group was conducted in Shanghai, China. Participants were recruited in the Department of Developmental and Behavioral Pediatrics in Xinhua Hospital between April 15, 2020 and March 19, 2021. The study was reviewed and approved by the Institutional Review Board of Xinhua Hospital (No: XHEC-C-2019-076). Informed consent was obtained from all parents after they were told about the study procedures and agreed to participate. Participation was entirely voluntary, and all participants had the right to withdraw at any time.

### Participants

**Figure 1:**
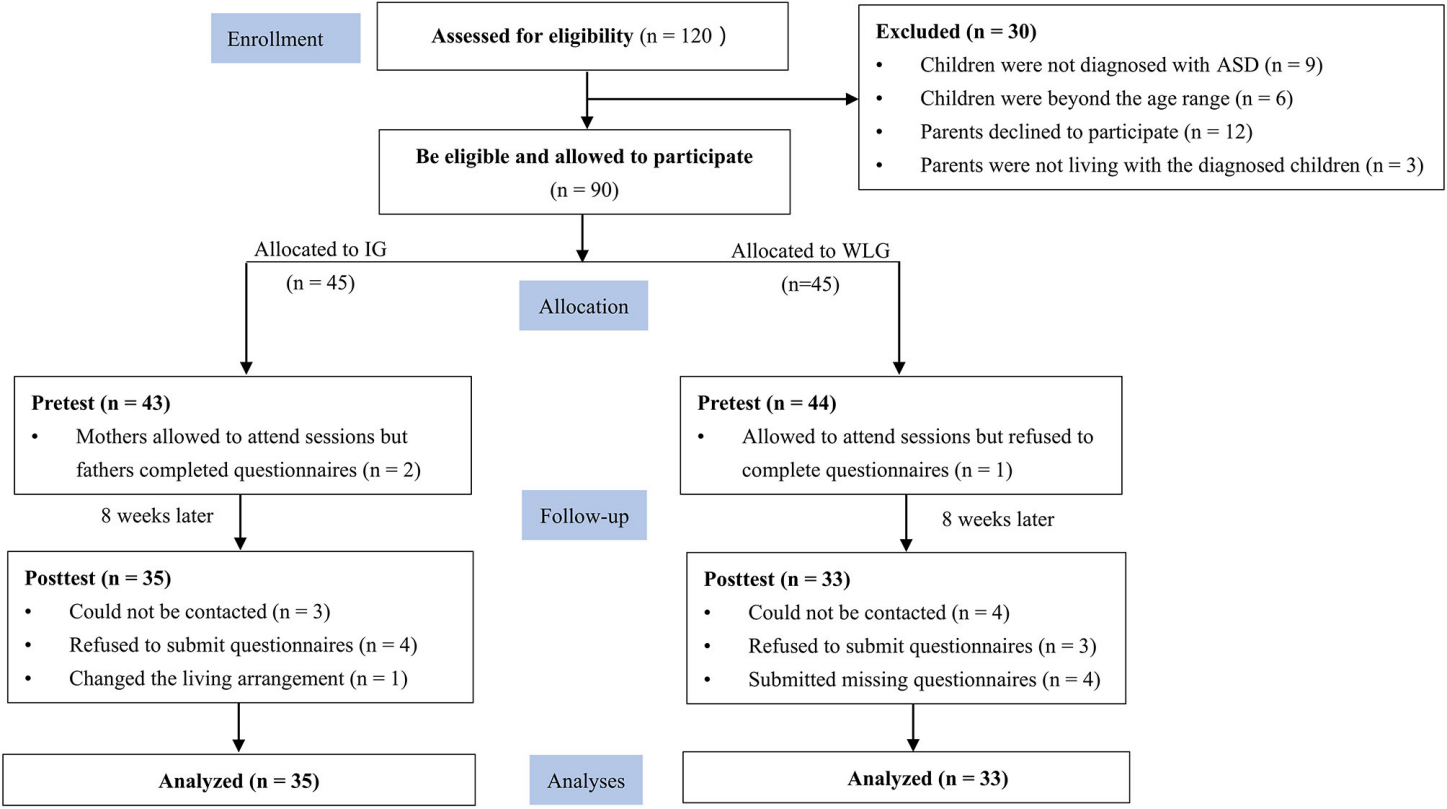
CONSORT diagram of the study flow. CONSORT, Consolidated Standards of Reporting Trials; ASD, autism spectrum disorder; IG, intervention group; WLG, waitlist group.

The present study recruited parent-child dyads. Participants were eligible if (a) children had a diagnosis of ASD according to the Diagnostic and Statistical Manual of Mental Disorders, Fifth Edition (DSM-V); (b) children were aged 2–6 years; (c) parents were aged 20–50 years; (d) parents had the internet access to online sessions and surveys; and (e) parents were living with the diagnosed children during the intervention period. Participants were excluded if (a) children were diagnosed with other mental disorders, physical diseases, or serious genetic abnormalities (e.g., traumatic brain injury and epilepsy requiring additional medical treatments); (b) parents were diagnosed with other mental diseases that may affect their participation (e.g., depression and dyslexia); (c) parents were non-biological parents; and (d) parents had more than one child diagnosed with ASD. The participant flowchart is presented in Figure 1.

An independent-samples *t*-test in the PASS 15.0 Software (NCSS Corp, Utah) was used to calculate the sample size. Based on a previous study (23), we assumed that the primary outcome (Social Responsiveness Scale, SRS) at baseline was comparable between the intervention and control groups. The posttest mean (M) score on the SRS was 67.5 for the intervention group and 77.78 for the control group, with an equal standard deviation (SD) of 14, power of 0.8, an alpha of 0.05, two-tailed, and a medium effect size (Cohen's *d* = 0.5). Thus, a total of 62 participants (31 per group) were needed to obtain statistical significance. We estimated an attrition rate of 20%, and finally recruited 90 participants.

### Procedures

Children's ASD diagnoses were made by developmental and behavioral pediatricians according to the DSM-V. Before being accepted into the study, ASD diagnoses were further verified by psychologists using the Childhood Autism Rating Scale (CARS) (27) and the Autism Diagnosis and Observation Scale (ADOS) (28). Recruitment leaflets that contained a detailed description of our research protocol were distributed to parents whose children had a confirmed ASD and who had registered for Project ImPACT (*n* = 120). The eligible participants who agreed to participate were recruited (*n* = 90). According to the order of enrolment, eligible parent-child dyads were allocated to either the intervention (IG; *n* = 45) or waitlist group (WLG; *n* = 45). Parents in the IG immediately received the online-delivered Project ImPACT program, and the WLG received the same program with a delay when the IG had completed all the sessions. Participants in both groups received treatment as usual during the research period. Links to online surveys were sent to all parents shortly before the beginning of the first session (T1) and 8 weeks later (T2). Those who did not complete the pretest questionnaires were not invited to submit the posttest questionnaires. The research assistant conducted the T1 and T2 assessments, and each lasted ~40 min. Clinicians, psychologists, and therapists were blinded to treatment allocation.

### Intervention Program

Given the long waitlist for early intervention services in China and the restrictions on face-to-face intervention services during the COVID-19 pandemic, some cultural adaptations to the Project ImPACT program were made to fit with the domestic medical practice. First, the original 12 weeks of sessions with 1 h per week were adapted into 8 weeks of 1.5-h sessions. Second, all sessions were delivered remotely in real-time. Third, group coaching was adopted (usually 8–10 parents per group) and WeChat group was created for on-line answer between sessions. Parents in the IG immediately received the online-delivered Project ImPACT program, with one session per week and 8 sessions in total. Each session was delivered through Tencent Meeting Application (Tencent Corp, Shanghai) and lasted ~1.5 h. To maintain therapist fidelity across different groups, all sessions were delivered by a certified psychological therapist from our research team, who received intensive training from the intervention developer through remote training. Session delivery was supervised by a senior administrator regularly. Parents in each group were managed in separate WeChat groups, in which an administrator, therapist, quality control supervisor, and research assistant were allocated. The training manual (Chinese version) was mailed to parents 1 week before the first session began. During the intervention period, parents in the WLG did not receive the Project ImPACT program until parents in the IG had finished all of the sessions. Parents and children in both groups received treatment as usual, which included various intervention services available in the communities.

The first session primarily involved goal setting and arrangement of the home environment. The second to eighth sessions began with a review of the content from the previous session, followed by an examination and evaluation of homework, didactic instruction of new strategies, a summary of the session, homework assignments, and finally ended with a question and answer (Q&A) section. The detailed contents of the 8-week Project ImPACT sessions are outlined in Supplementary Table S1. Before each session, the research assistant reminded parents to preview the corresponding chapters, and a live link to the cloud conference room was generated using the Tencent Meeting Application. Link was sent to the WeChat groups, and parents could attend the sessions by clicking the link. Between sessions, parents were allowed to ask course-related questions and upload video clips of parent-child interactions in the WeChat groups. The therapist answered their questions, watched the home videos to detect any areas for improvement and provided individualized feedback to parents on a daily basis.

### Measures

#### Demographic Questionnaire

General data on child and parent characteristics were collected before treatment allocation. Child characteristics included age, gender, only child status, language level, diagnosis subtype, and additional intervention services received. Parent characteristics included parental role, age, marital status, educational level, employment status and residence.

#### Social Responsiveness Scale

The Social Responsiveness Scale (SRS) is a parent-reported questionnaire that measures the severity of children's social deficits associated with ASD in natural social situations (29). It consists of 65 items that are divided into five dimensions: social awareness, social cognition, social communication, social motivation, and autistic mannerisms. Each item is rated on a 4-point Likert scale, and a higher score indicates greater severity of social impairment. The Cronbach's α coefficient for the SRS total scale was 0.94 and the subscales ranged from 0.55 to 0.85 at pretest. The corresponding Cronbach's α coefficients at posttest were 0.93 for the total scale and 0.56–0.87 for the subscales.

#### Autism Treatment Evaluation Checklist

The Autism Treatment Evaluation Checklist (ATEC) is a self-reported scale designed to assess the change in autism severity in intervention studies (30). It is composed of four subscales: language/speech/communication, sociability, sensory/cognitive awareness, and health/physical/ behavior. In this study, we adopted the first two subscales only, which mainly reflect the social function of children. Each item of the sociability subscale is rated on a 3-point Likert scale, with total scores ranging from 0 to 40. The items of the language/speech/communication subscale are reverse-scored, with higher scores indicating greater severity of autistic symptoms. In this study, the Cronbach's α coefficients at pretest were 0.95 for the language subscale and 0.90 for the sociability subscale. The corresponding Cronbach's α coefficients at posttest were 0.94 and 0.91.

#### Parenting Stress Index-Short Form

The Parenting Stress Index-Short Form (PSI-SF) is a 36-item self-reported questionnaire that measures stress related to parenting roles (31). It consists of three subscales: parental distress, parent-child dysfunctional interaction, and difficult child. Each subscale contains 12 items rated on a 5-point Likert scale. The PSI-SF yields a total score ranging from 36 to 180, with a higher score indicating a greater level of parenting stress. A score above 90 indicates a clinical level of stress, and a score of 90 or below represents a low level of stress. The Cronbach's α coefficients for the PSI-SF in this study indicated excellent internal consistency on both pretest and posttest measures (Pretest Cronbach's α coefficient was 0.94 and posttest Cronbach's α coefficient was 0.89).

#### Parental Self-Efficacy of Competence Scale

The Parental Self-efficacy of Competence Scale (PSOC) was developed by Gibaud-Wallston (32), which has been used widely in ASD-related research to assess parents' belief and confidence in fostering their child's development. It is a 17-item scale that consists of two dimensions: eight items in the efficacy subscale and nine items in the satisfaction subscale. Each item is rated on a 6-point Likert scale and items in the satisfaction subscale are reverse-scored. A higher score represents higher parental self-efficacy. A previous study validated the psychometric properties of the PSOC in Chinese mothers, which demonstrated good internal consistency (33). In this study, the Cronbach's α coefficients were acceptable at pretest (Efficacy: 0.72; Satisfaction: 0.73) and posttest (Efficacy: 0.75; Satisfaction: 0.76).

### Data Analysis

The SPSS 24.0 software (IBM Corp; Armonk) was used for data analyses. The normality of the continuous variables was assessed using the Shapiro-Wilk test. Continuous variables that were normally distributed were expressed as means and standard deviations (*M* ± SD), and if not normally distributed, as medians and interquartile ranges (IQR). Categorical variables were presented as frequencies (*N*) and percentages (%). Independent-samples *t-*tests, chi-square tests, and Mann-Whitney *U-*tests were performed to compare the demographic characteristics between groups. Paired-samples *t*-tests were used to compare the differences between the pretest and posttest outcomes Group differences in outcome measures following the intervention program were explored using one-way analyses of covariance (ANCOVA), and η^2^ was calculated to evaluate the effect size of these difference (η^2^ ≥ 0.01: small effect, η^2^ ≥ 0.06: medium effect, η^2^ ≥ 0.14: large effect) (34).

## Results

### Sample Characteristics and Group Differences at Baseline

Among the initial 120 parent-child dyads assessed for eligibility, 90 eligible dyads were recruited, and 68 were included in the final analyses, which resulted in a retention rate of 75.6%. Of the 22 parent-child dyads excluded from the final analyses, seven parents could not be contacted, eight refused to submit questionnaires, six submitted unqualified questionnaires, and one changed their living arrangement. The mean age of the 68 children was 3.67 years (SD = 0.99). Seventy percent of the children were from one-child families, and the majority (82.4%) were boys. Nearly one-third of children (27.9%) were non-verbal, and exactly half spoke single words or phrases. The subtypes of diagnosis were autism for 65 children (95.6%) and pervasive developmental disorder not otherwise specified (PDD-NOS) for three children (4.4%). The mean CARS score for the total sample was 36.20 (SD = 3.11). Of these, 39 children (57.4%) had a mild-to-moderate level of symptoms, and the remaining 29 (42.6%) had a severe level of symptoms (i.e., a total score > 36 and a rating of ≥ 3 on at least five items). In regard to additional intervention services, 19.1% of children received no other services, and those remaining received either family (26.5%) or institutional interventions (54.4%). The 68 participating parents consisted of 56 mothers (82.4%) and 12 fathers (17.6%), of whom 66 (97.1%) were married, with a mean age of 34.12 years (SD = 4.71). The majority had a college degree or above (77.94%) and 70.59% of them were employed. Sixty-two (91.2%) parents lived in urban areas. There were no significant group differences in any of these child and parent characteristics between groups (Supplementary Table S2). Sample characteristics were also compared for the completers and non-completers, and no significant difference was detected (Supplementary Tables S3, S4).

### Changes in Child and Parent Outcomes Within Groups

Following 8 weeks of intervention, there was a significant improvement in children's social communication skills [*t*_(34)_ = 3.56, *P* = 0.001], language [*t*_(34)_ = 3.43, *P* = 0.002], sociability [*t*_(34)_ = 5.04, *P* < 0.001], parents' perceived competence in the parental role [*t*_(34)_ = −2.51, *P* = 0.017], and parenting stress [*t*_(34)_ = 4.25, *P* < 0.001], but the change in the parental satisfaction domain of the PSOC was not significant [*t*_(34)_ = 0.56, *P* = 0.58]. In contrast, children and parents in the WLG did not show significant improvements during the intervention period in terms of children's social communication skills [*t*_(32)_ = −0.30, *P* = 0.76], language [*t*_(32)_ = 0.68, *P* = 0.50], parenting stress [*t*_(32)_ = 0.60, *P* = 0.55] or parental self-efficacy [*t*_(32)_ = 0.33, *P* = 0.74], except for the sociability domain of the ATEC [*t*_(32)_ = 2.20, *P* = 0.04]. Results of the comparisons within groups are provided in the Supplementary Material (Table 1).

**Table 1:** Comparison of child and parent outcomes within groups.

**Variables**	**IG (*****n*** **=** **35)**		***t_**1**_***	**WLG (*****n*** **=** **33)**		***t_**2**_***
	**Pretest**	**Posttest**		**Pretest**	**Posttest**	
**SRS**						
Social awareness	11.54 ± 3.09	11.17 ± 2.42	0.83	11.85 ± 2.60	12.12 ± 3.24	−0.51
Social cognition	18.11 ± 4.05	16.69 ± 3.46	2.61^*^	17.39 ± 4.59	18.61 ± 4.92	−1.62
Social communication	33.11 ± 8.35	29.66 ± 8.50	3.10^**^	33.03 ± 8.65	32.42 ± 9.33	0.48
Social motivation	15.17 ± 4.88	13.49 ± 3.81	3.28^**^	14.94 ± 4.94	14.15 ± 4.47	1.10
Autistic mannerisms	14.49 ± 7.03	12.74 ± 5.19	1.99	12.91 ± 6.80	13.85 ± 6.18	−0.96
Total scores	92.43 ± 22.85	83.74 ± 18.3	3.56^**^	90.12 ± 23.63	91.15 ± 24.74	−0.30
**ATEC**						
Language	13.06 ± 8.12	11.63 ± 7.59	3.43^**^	13.23 ± 6.70	12.70 ± 7.32	0.68
Sociability	18.03 ± 7.68	13.63 ± 6.82	5.04^**^	19.41 ± 6.73	16.85 ± 7.46	2.20^*^
Total scores	31.09 ± 12.37	25.26 ± 12.16	5.36^**^	32.64 ± 11.18	29.55 ± 11.92	2.20^*^
**PSI-SF**						
PD	35.77 ± 8.18	33.17 ± 7.39	2.54^*^	34.64 ± 11.02	32.97 ± 10.34	1.12
PCDI	31.49 ± 9.10	28.03 ± 6.45	3.50^**^	28.85 ± 6.55	28.82 ± 7.22	0.04
DC	35.80 ± 8.54	32.31 ± 6.18	3.41^**^	35.91 ± 7.48	36.18 ± 8.32	−0.28
Total scores	103.06 ± 22.21	93.51 ± 16.84	4.25^**^	99.39 ± 19.34	97.97 ± 21.09	0.60
**PSOC**						
Satisfaction	31.00 ± 6.05	30.54 ± 6.02	0.56	32.73 ± 8.04	31.79 ± 7.13	0.68
Efficacy	27.66 ± 6.30	30.49 ± 4.06	−2.51^*^	29.39 ± 5.76	29.85 ± 5.47	−0.49
Total scores	58.66 ± 9.78	61.03 ± 7.45	−1.93	62.12 ± 7.47	61.64 ± 5.25	0.33

*IG, intervention group; WLG, waitlist group; SRS, social responsiveness scale; ATEC, autism treatment evaluation checklist; PSI-SF, parenting stress index-short form; PD, parental distress; PCDI, parent-child dysfunctional interaction; DC, difficult child; PSOC, parental self-efficacy of competence scale; t_1_, within group comparison for the IG; t_2_, within group comparison for the WLG*.

* *P < 0.05*.

** *P < 0.01*.

### Differences in Child and Parent Outcomes Between Groups

Results of the ANCOVA showed that the baseline SRS score had a significant effect on children's social impairment at posttest [*F*_(1, 65)_ = 66.64, *P* < 0.001, partial η^2^ = 0.51], and there was also a significant effect of treatment allocation on children's social communication skills [*F*_(1, 65)_ = 5.76, *P* = 0.019, partial η^2^ = 0.08], where children in the IG showed greater improvement than those in the WLG (Table 2). Adjusted mean SRS scores at posttest in the IG and WLG were 83.0 and 91.94, respectively. When we examined the effect of intervention on the SRS subscales, we found a significant effect of treatment allocation on social cognition [*F*_(1, 65)_ = 8.09, *P* = 0.006, partial η^2^ = 0.11].

**Table 2:** Analyses of covariance for child and parent variables.

**Source**	**SS**	**df**	**MS**	***F***	***P***	**η2**
**Child's social impairment**						
T1 SRS	15,678.01	1	15,678.01	66.64	<0.001	0.51
Group	1,353.91	1	1,353.91	5.76	**0.019**	0.08
**Child's sociability**						
T1 ATEC_sociability	493.29	1	493.29	18.15	<0.001	0.22
T1 SRS	168.62	1	168.62	6.21	0.015	0.09
Group	125.88	1	125.88	4.63	**0.035**	0.07
**Parenting stress**						
T1 PSI-SF	14,346.83	1	14,346.83	97.91	<0.001	0.60
Group	836.09	1	836.09	5.71	**0.020**	0.08
**Parents' perceived competence in the parental role**						
T1 PSOC_efficacy	262.10	1	262.10	13.93	<0.001	0.18
Group	95.30	1	95.30	5.06	**0.028**	0.07

*SS, sum of squares; MS, mean square; SRS, social responsiveness scale; ATEC, autism treatment evaluation checklist; PSI-SF, parenting stress index-short form; PSOC, parental self-efficacy of competence scale; η2 ≥ 0.01, small effect; η2 ≥ 0.06, medium effect; η2 ≥ 0.14, large effect. P values in bold indicate a statistically significant difference (P < 0.05)*.

There was no significant effect of group allocation on children's language [*F*_(1, 64)_ = 1.02, *P* = 0.315, partial η^2^ = 0.02], which was mainly predicted by the language level at baseline [*F*_(1, 64)_ = 224.51, *P* < 0.001, partial η^2^ = 0.78]. For sociability, results showed that the baseline level of sociability [*F*_(1, 64)_ = 18.15, *P* < 0.001, partial η^2^ = 0.22] and SRS score [*F*_(1, 64)_ = 6.21, *P* = 0.015, partial η^2^ = 0.09] had a significant effect on posttest sociability. Furthermore, there was a significant effect of group allocation on post-intervention sociability [*F*_(1, 64)_ = 4.63, *P* = 0.035, partial η^2^ = 0.07], with children in the IG showing greater improvement than those in the WLG (Table 2). The adjusted sociability score of the IG was 13.85 after the intervention compared with 16.61 of the WLG.

The results of the ANCOVA demonstrated that parenting stress after the intervention was mainly predicted by the stress level at baseline [*F*_(1, 65)_ = 97.91, *P* < 0.001, partial η^2^ = 0.60]. Moreover, the effect of treatment allocation on parenting stress was also significant [*F*_(1, 65)_ = 5.71, *P* = 0.02, partial η^2^ = 0.08], where parents in the IG showed a lower level of stress after the intervention (Table 2). The adjusted PSI-SF score was 99.30 in the WLG, whereas that of the IG was 92.26 after the intervention. When we examined the effect of intervention on the subscales, we found a significant effect on the parent-child dysfunction interaction [*F*_(1, 65)_ = 4.67, *P* = 0.03, partial η^2^ = 0.07] and difficult child [*F*_(1, 65)_ = 9.30, *P* = 0.003, partial η^2^ = 0.13]. It should be noted that the proportion of parents with clinically significant stress in the IG decreased from 71.4 to 60%, whereas in the WLG, there was an increase from 69.7 to 72.7% during the research period.

We did not find a significant group difference in parents' satisfaction with the parenting role after the intervention [*F*_(1, 65)_ = 0.07, *P* = 0.79, partial η^2^ = 0.001], which was mainly predicted by the satisfaction level at baseline [*F*_(1, 65)_ = 27.59, *P* < 0.001, partial η^2^ = 0.30]. The perceived competence in the parental role at baseline had a significant effect on the posttest competence level [*F*_(1, 64)_ = 13.93, *P* < 0.001, partial η^2^ = 0.18]. In addition, there was a significant effect of group allocation on posttest competence [*F*_(1, 64)_ = 5.06, *P* = 0.028, partial η^2^ = 0.07], where parents in the IG showed greater improvement than those did in the WLG (Table 2).

### Feasibility of the Online-Delivered Project ImPACT Program

Among the parents in the IG, 87.5% evaluated the schedule of sessions to be reasonably arranged, and 98.2% considered the contents well prepared and precisely targeted. Three-quarters of parents reported that could deal with the homework between sessions and believed that the homework facilitated their mastery of strategies and techniques. Furthermore, 98.2% of parents reported that they received instant feedback from the therapist and that the feedback helped them solve problems.

## Discussion

This pilot waitlist control study evaluated the effectiveness of an 8-week, online-delivered Project ImPACT program for families of children with ASD in China during the COVID-19 pandemic. Findings from our study showed positive changes in child and parent outcomes following the intervention program. Consistent with our hypothesis, the online-delivered Project ImPACT program significantly improved child's social communication skills compared with that of the WLG, especially in the domains of social cognition. Moreover, this was further supported by the analysis of the sociability subscale of the ATEC. For parents, there was a significant reduction in parenting stress among those in the IG compared with their counterparts in the WLG, and the reduction was especially marked in the domains of parent-child dysfunctional interaction and difficult child. Furthermore, we found an obvious reduction in the proportion of parents with clinically significant stress in the IG. In contrast, the proportion of parents with clinically significant stress in the WLG showed a slight increase. Moreover, parents in the IG showed a significant improvement in perceived competence in the parental role, whereas that of the parents in the WLG remained relatively stable. There were no significant improvements in children's language or parents' satisfaction with the parenting role after the intervention. Parents rated the program highly in terms of curriculum schedule, session content, homework assignments, and therapist feedback. Taken together, these results provide preliminary evidence for the effectiveness of the online-delivered Project ImPACT program for families of children with ASD in China during the COVID-19 pandemic.

The pervasive impairment of social communication is one of the core symptoms for children with ASD, and the online-delivered Project ImPACT program specifically targets the critical domains of children's social development. As noted in a previous study (24), children who receive 12 weeks of the Project ImPACT program demonstrate significantly greater gains in social communication skills compared with those in the community. Although the intervention program in the current study was adapted to 8 sessions and delivered remotely, our results corresponded to those of the previous study. There are several potential reasons for the positive changes. First, *via* the Project ImPACT online training, parents consciously adjusted their communication strategies to interact with children more efficiently, and they tended to be more sensitive and responsive to their child's social cues, which increased the opportunities for meaningful parent-child interactions. Parents who act synchronously with their children's focus and intentions are more likely to enhance their children's social communication skills (35). Second, the improvement in children's social communication skills may be attributed to the active involvement of parents. Parents are usually the family members who spend the most time with children, and this is particularly true during the COVID-19 pandemic. Coaching parents to be the mediators of social skill interventions for children allows the training to be integrated into daily life and routine activities, which maximizes the exposure of children to a favorable communicative environment (36). Finally, all parents in our study were managed using WeChat groups, which provided a platform for reinforced learning and opportunities for booster training between sessions. Parents were permitted to ask questions and upload video clips of parent-child interactions to the groups, and the therapist offered feedback on an individual basis. The support and supervision from professionals may motivate parents to constantly engage in practicing social interactions with their children.

We also observed a significant reduction in parenting stress and an increase in perceived competence in the parental role following the intervention. It is well established that parents of children with ASD experience an elevated level of stress compared with those of typically developing children and children with other developmental disabilities (37, 38). Moreover, these parents seldom obtain emotional reciprocity with their children during daily interactions; thus, they are susceptible to emotional frustration and low parenting self-efficacy (39). As an enabling process, the Project ImPACT program equipped parents with strategies and techniques for social communication, and parents were empowered to flexibly and effectively interact with their children during daily activities (22, 24, 26), which increased parents' confidence in parental tasks and reduced the pressure on parental roles. The Project ImPACT program delivered online encouraged parents to utilize various resources and maximize multimedia functions (e.g., interactive learning, group discussion, video telephone, and online Q&A). Additionally, parents could share disease-related information, parenting experience, and emotional distress in the WeChat group. The cultivation of a peer support network among parents may facilitate the improvement of parental psychological wellbeing and increase their confidence in the parental role (40).

Although we detected a significant effect of the online-delivered Project ImPACT program on children's social communication skills and parenting stress, it is worth noting that the level of social impairment at baseline was also a significant predictor of social communication skills after the 8 weeks of intervention. Similarly, baseline parenting stress also positively predicted post-intervention stress levels. Following the intervention program, children's language and parents' satisfaction with the parenting role did not show a significant difference in either group. Two implications are informed for future course optimization: (1) professionals should pay special attention to children with initially severe social impairment or a low language level, who may benefit more from customized sessions with a longer duration and booster training and (2) some parent support programs aimed at stress management and psychological adjustment can be integrated into current sessions.

Parent feedback showed an optimistic attitude toward the online-delivered and group-based Project ImpACT program. Families of children with ASD in our outpatient department come from across the country, and more than half of our study cohort (62.38%) are living outside of Shanghai. Parents of children with ASD tend to be young and thus are receptive to new delivery models of medical services. In addition, there is a long waitlist for early intervention program. All these characteristics laid the foundation for cultural adaptation of Project ImPACT program. We introduced Project ImPACT and delivered remotely in a group format. The Project ImPACT program delivered online allowed for maximum inclusion of families who have limited access to on-site training programs. In fact, distance training for parents of children with ASD has increased in recent years, particularly during the COVID-19 pandemic. Parent training delivered remotely has the potentials to enhance parental knowledge, increase parent intervention fidelity, and improve the social behaviors and communication skills of children with ASD (19). The use of telehealth programs to train parents of children with ASD in evidence-based intervention techniques is a promising area of research in future studies. In addition, we adopted group coaching due to the long waitlist and limited early intervention services. It is a highly recommended practice in resource-limited areas, because sessions delivered in a group format is not only cost and time effective, but also facilitate the building support network among parents.

## Limitations

Although the online-delivered Project ImPACT program show preliminary effectiveness for children with ASD and their parents, parents were allowed to access usual care in the community for ethical consideration, which makes it necessary to be cautious of the conclusion. Meanwhile, there are several limitations to this study. First, a lack of randomization in treatment allocation and the relatively large rate of attrition weakened the methodological quality of the study. Second, the intervention period lasted 8 weeks, and no long-term follow-up data were collected; therefore, we could not evaluate maintenance effects. For future studies, randomized controlled studies with larger sample sizes and longer follow-up periods are recommended to provide more solid conclusions for the program. Third, the outcome measures in this study were parent-reported. To avoid reporter bias, more reliable measures (e.g., video coding, clinical interviews, and physical assessments) were recommended in future studies. Finally, we did not collect information on parents' implementation of techniques at home (e.g., fidelity, facilitators, and barriers) and the details of parental usage of online Q&A (e.g., video unloading and therapist feedback). Thus, qualitative studies may offer a deeper insight into parental perceptions of the program and the process of changes from a different perspective.

## Conclusions

The 8-week, online-delivered Project ImPACT program is an effective intervention program for families of children with ASD in China during the COVID-19 pandemic. It has the potential to improve children's social impairment, alleviate parenting stress, and increase parents' perceived competence in the parental role. Given the scarcity of early intervention services for children with ASD in low-resource areas, the Project ImPACT program delivered online may be a promising program to promote intervention accessibility and improve health outcomes for children with ASD and their parents.

## Data Availability Statement

The raw data supporting the conclusions of this article will be made available by the authors, without undue reservation.

## Ethics Statement

The studies involving human participants were reviewed and approved by Institutional Review Board of Xinhua Hospital. Written informed consent to participate in this study was provided by the participants' legal guardian/next of kin.

## Author Contributions

FēL was responsible for the design of research, collection and analyses of data, and drafting of the manuscript. DW was responsible for the delivery of all sessions and on-line answer in the WeChat group. FR, LS, MXue, and JY were involved in the organization and arrangement of sessions. FeL and LJ provided guidance on the design of the study and supervised the implementation of the research. MXu was in charge of the quality control of session delivery and coordinated between our research team and the Project ImPACT developers. YT, LZha, XL, and MT were involved in participant recruitment, data preparation, statistical analyses, and manuscript revision. LZho was responsible for the language quality of the manuscript. All authors contributed to the article and approved the submitted version.

## Funding

This study was supported by grants from the National Natural Science Foundation of China (71874107, 82125032, 81930095, and 81761128035), Shanghai Municipal Health Commission (2019SY068), the Science and Technology Commission of Shanghai Municipality (19410713500 and 2018SHZDZX01), the Shanghai Municipal Commission of Health and Family Planning (GWV-10.1-XK07, 2020CXJQ01, 2018YJRC03, and 2018BR33), and the Guangdong Key Project (2018B030335001).

## Conflict of Interest

The authors declare that the research was conducted in the absence of any commercial or financial relationships that could be construed as a potential conflict of interest.

## Publisher's Note

All claims expressed in this article are solely those of the authors and do not necessarily represent those of their affiliated organizations, or those of the publisher, the editors and the reviewers. Any product that may be evaluated in this article, or claim that may be made by its manufacturer, is not guaranteed or endorsed by the publisher.
