# Supplementary material for: Effectiveness of Online-Delivered Project ImPACT for Children With ASD and Their Parents: A Pilot Study During the COVID-19 Pandemic
Source: Front Psychiatry. 2022 Mar 24;13:806149. doi: 10.3389/fpsyt.2022.806149 (PMC8987566; doi:10.3389/fpsyt.2022.806149)
Supplement: Supplementary file 1 [file Data_Sheet_1.pdf]

Table S1 Overview of 8-week Project ImPACT sessions

Session	Theme	Contents
1	Get Started	The Introduction of Program
		Goal Setting
		Set up the Home Environment
2	Focus on Your Child	Follow Child’s Lead
		Imitate Your Child
3	Adjust Your Communication	Use Animation
		Model and Expand Communication
4	Create Opportunities	Playful Obstruction
		Balanced Turns
		Communicative Temptations
5	Teach New Communication Skills (1)	Prompts and Rewards
		Prompts for Understanding Communication
6	Teach New Communication Skills (2)	Prompts for Using Communication
7	Teach New Imitation and Play Skills	Prompts for Imitation
		Prompts for Expanding Play
8	Shape the Interaction and Move Forward	Shape the Interaction
		Use Project ImPACT in the Community
		Introduce Plan for Continued Success

Table S2 Demographic characteristics of participants in each group

Categories	IG (n = 35)	WLG (n = 33)	Statistics	*P* value
**Child demographics**				
Age, *M* ± *SD*	3.87 ± 0.98	3.47 ± 0.98	−1.69	0.10[a]
Gender				
Male	30 (85.7%)	26 (78.8%)	0.56	0.45[b]
Female	5 (14.3%)	7 (21.2%)		
An only child or not				
Yes	26 (74.3%)	21 (63.6%)	0.90	0.34[b]
No	9 (25.7%)	12 (36.4%)		
Language level				
Non-verbal	10 (28.6%)	9 (27.3%)	0.71	0.70[b]
Single words or phrase	16 (45.7%)	18 (54.5%)		
Sentence	9 (25.7%)	6 (18.2%)		
Diagnosis				
Autism	34 (97.1%)	31 (93.9%)	/	0.61[c]
PDD-NOS	1 (2.9%)	2 (6.1%)		
CARS, M±SD	36.34±3.33	36.05±2.90	−0.38	0.71[a]
Severity of symptoms				
Mild-to-moderate	19 (54.3%)	20 (60.6%)	0.28	0.60[b]
Severe	16 (45.7%)	13 (39.4%)		
Additional interventions (type)				
No intervention	5 (14.3%)	8 (24.2%)	1.55	0.46[b]
Family interventions	11 (31.4%)	7 (21.2%)		
Institutional interventions	19 (54.3%)	18 (54.5%)		
**Parent characteristics**				
Role				
Mothers	27 (77.1%)	29 (87.9%)	1.35	0.25[b]
Fathers	8 (22.9%)	4 (12.1%)		

Age, $M \pm SD$	34.54 ± 4.83	33.67 ± 4.60	–0.77	0.45[a]
Marital status				
Married	35 (100%)	31 (93.9%)	/	0.23[c]
Separated	0 (0%)	2 (6.1%)		
Education				
Below college degree	9(25.7%)	6(18.2%)	0.45	0.56 [b]
College or above degree	26(74.3%)	27(81.8%)		
Employment status				
Employed	24(68.6%)	24(72.7%)	0.71	0.79 [b]
Unemployed	11(31.4%)	9(27.3%)		
Residence				
Rural	2 (5.7%)	4 (12.1%)	/	0.42 [c]
Urban	33 (94.3%)	29 (87.9%)		
Monthly family income				
2-4 thousand	0(0%)	2(6.1%)	7.63	0.27
4-6 thousand	2(5.7%)	4(12.1%)		
6-8 thousand	4(11.4%)	1(3.0%)		
8-10 thousand	5(14.3%)	1(3.0%)		
10-20 thousand	11(31.4%)	9(27.3%)		
20-50 thousand	7(20.0%)	8(24.2%)		
>50 thousand	6(17.1%)	8(24.2%)		

Note. IG: intervention group; WLG: waitlist group; PDD-NOS: pervasive developmental disorder not otherwise specified; CARS: Childhood Autism Rating Scale; *M*: mean; *SD*: standard deviation; [a] *t* test; [b] Chi-square test; [c] Fisher's exact test.

Table S3 Demographic characteristics of completer and noncompleter

**Categories**	**Completer (n = 68)**	**Noncompleter (n = 22)**	**Statistics**	***P* value**
**Child demographics**				
Age, *M* ± *SD*	3.67±0.99	3.62±3.11	0.36	0.722 [a]
Gender				
Male	56(82.35%)	18(81.82%)	/	1.000 [c]
Female	12(17.65%)	4(18.18%)		
An only child or not				
Yes	47(69.12%)	14(63.64%)	0.63	0.793 [b]
No	21(30.88%)	8(36.36%)		
Language level				
Non-verbal	19(27.94%)	2(9.09%)	/	0.133 [c]
Single words or phrase	34(50.0%)	16(72.73%)		
Sentence	15(22.06%)	4(18.18%)		
Diagnosis				
Autism	65(95.59%)	19(86.36%)	/	0.154 [c]
PDD-NOS	3(3.33%)	3(13.64%)		
CARS, M±SD	36.20±3.11	35.45±2.88	1.00	0.32 [a]
Severity of symptoms				
Mild-to-moderate	39(57.35%)	14(63.64%)	0.60	0.629 [b]
Severe	29(42.65%)	8(36.36%)		
Additional intervention				
No intervention	13(19.12%)	3(13.64%)	/	0.840 [c]
Family interventions	18(26.47%)	5(22.73%)		
Institutional interventions	37(54.41%)	14(63.64%)		
**Parent characteristics**				
Role				
Mothers	56(82.35%)	16(59.09%)	/	0.364 [c]
Fathers	12(17.65%)	6(40.91%)		

Age, $M \pm SD$	34.12±4.71	35.36±4.89	1.39	0.167 [a]
Marital status				
Married	66(97.06%)	22(100%)	/	1.000 [c]
Separated	2(2.92%)	0(0%)		
Education				
Below college degree	15(22.06%)	4(18.18%)	/	1.000 [c]
College or above degree	53(77.94%)	18(81.82%)		
Employment status				
Employed	48(70.59%)	14(63.64%)	0.54	0.6 [b]
Unemployed	20(29.41%)	8(36.36%)		
Residence				
Rural	6(8.82%)	0(0%)	/	0.33 [c]
Urban	62(91.18)	22(100%)		
Monthly family income				
2-4 thousand	2(2.9%)	0(0%)	9.498	0.147 [b]
4-6 thousand	6(8.8%)	0(0%)		
6-8 thousand	5(7.4%)	2(9.1%)		
8-10 thousand	6(8.8%)	3(13.6%)		
10-20 thousand	20(29.4%)	10(45.45%)		
20-50 thousand	15(22.1%)	7(31.82%)		
>50 thousand	14(20.6%)	0(0%)		

Note. PDD-NOS: pervasive developmental disorder not otherwise specified; CARS: Childhood Autism Rating Scale; *M*: mean; *SD*: standard deviation. [a] *t* test; [b] Chi-square test; [c] Fisher's exact test.

Table S4 Outcome measures of the completer and noncompleter at baseline

Variables	Completer (n = 68)	Noncompleter (n = 22)	Statistics	*P* value
**SRS**				
Social awareness	11.69±2.85	11.68±2.72	0.014	0.989
Social cognition	17.76±4.30	19.45±5.54	–1.489	0.140
Social communication	33.07±8.43	32.95±9.09	0.056	0.955
Social motivation	15.06±4.87	14.86±5.40	0.159	0.874
Autistic mannerisms	13.72±6.92	14.55±7.11	–0.483	0.630
Total scores	91.31±23.09	93.50±26.70	–0.372	0.711
**ATEC**				
Language	13.14±7.70	12.32±6.35	0.451	0.653
Sociability	18.70±7.50	17.05±8.17	0.870	0.387
Total scores	31.8412.21	29.36±11.95	0.824	0.413
**PSI-SF**				
PD	35.22±9.61	33.73±8.31	0.654	0.515
PCDI	30.21±8.02	29.73±6.47	0.254	0.80
DC	35.85±20.79	37.05±7.77	–0.613	0.547
Total scores	101.28±20.79	100.50±18.98	0.156	0.876
**PSOC**				
Satisfaction	31.84±28.50	32.23±6.55	–0.023	0.82
Efficacy	28.50±6.06	28.0±4.80	0.352	0.725
Total scores	60.34±8.84	60.23±8.51	0.052	0.959

Note. SRS: Social Responsiveness Scale; ATEC: Autism Treatment Evaluation Checklist; PSI-SF: Parenting Stress Index-Short Form; PD: parental distress; PCDI: parent-child dysfunctional interaction; DC: difficult child; PSOC: Parental Self-efficacy of Competence Scale; *P <0.05; **P <0.01

Table S5 Demographic characteristics of participants at baseline and exit in each group

Categories	IG		WLG		Statistics [#]	*P* value [#]
	Baseline (n=45)	Exit (n=35)	Baseline (n=45)	Exit (n=33)		
**Child demographics**						
Age, $M \pm SD$	3.80±0.88	3.87 ± 0.98	3.53±0.85	3.47 ± 0.98	−1.48	0.143 [a]
Gender						
Male	39 (86.7%)	30 (85.7%)	35 (77.8%)	26 (78.8%)	1.216	0.270 [b]
Female	6 (13.3%)	5 (14.3%)	10 (22.2%)	7 (21.2%)		
An only child or not						
Yes	32 (71.7%)	26 (74.3%)	29 (64.4%)	21 (63.6%)	0.458	0.499 [b]
No	13 (28.9%)	9 (25.7%)	16 (35.6%)	12 (36.4%)		
Language level						
Non-verbal	12 (26.7%)	10 (28.6%)	9 (20%)	9 (27.3%)	0.801	0.067 [b]
Single words or phrase	23 (51.1%)	16 (45.7%)	27 (40%)	18 (54.5%)		
Sentence	10 (22.2%)	9 (25.7%)	9 (20%)	6 (18.2%)		
Diagnosis						
Autism	41 (91.1%)	34 (97.1%)	43 (95.6%)	31 (93.9%)	/	0.677 [c]
PDD-NOS	4 (8.9%)	1 (2.9%)	2 (4.4%)	2 (6.1%)		
CARS, M±SD	36.02±3.32	36.34±3.33	36.02±2.81	36.05±2.90	−0.006	0.995 [a]
Severity of symptoms						
Mild-to-moderate	26 (57.8%)	19 (54.3%)	27 (60%)	20 (60.6%)	0.046	0.83 [b]
Severe	19 (42.2%)	16 (45.7%)	18 (40%)	13 (39.4%)		
Additional interventions (type)						
No intervention	7 (15.6%)	5 (14.3%)	9 (20%)	8 (24.2%)	1.513	0.469 [b]
Family interventions	14 (31.1%)	11 (31.4%)	9 (20%)	7 (21.2%)		
Institutional interventions	24 (53.3%)	19 (54.3%)	27 (60%)	18 (54.5%)		
**Parent characteristics**						
Role						
Mothers	34 (75.6%)	27 (77.1%)	35 (77.8%)	29 (87.9%)	0.062	0.803 [b]
Fathers	11(24.4%)	8 (22.9%)	10 (22.2%)	4 (12.1%)		

Age, *M* ± *SD*	34.71±5.14	34.54±4.83	34.13±4.37	33.67±4.60	–0.575	0.567 [a]
Marital status						
Married	45(100%)	35 (100%)	43 (95.6%)	31 (93.9%)	/	0.494 [c]
Separated	0 (0%)	0 (0%)	2 (4.4%)	2 (6.1%)		
Education						
Below college degree	10 (22.2%)	9(25.7%)	7 (15.6%)	6(18.2%)	0.653	0.419 [b]
College or above degree	35 (77.8%)	26(74.3%)	38 (84.4%)	27(81.8%)		
Employment status						
Employed	29 (64.4%)	24(68.6%)	33 (73.3%)	24(72.7%)	0.829	0.362 [b]
Unemployed	16 (35.6%)	11(31.4%)	12 (26.7%)	9(27.3%)		
Residence						
Rural	2 (4.4%)	2(5.7%)	4 (8.9%)	4 (12.1%)	/	0.677 [c]
Urban	43 (95.6%)	33 (94.3%)	41 (91.1%)	29 (87.9%)		
Monthly family income						
2-4 thousand	0 (0.0%)	0 (0%)	2 (4.4%)	2 (6.1%)	7.134	0.309 [b]
4-6 thousand	2 (4.4%)	2 (5.7%)	4 (8.9%)	4 (12.1%)		
6-8 thousand	4 (8.9%)	4 (11.4%)	3 (6.7%)	1 (3.0%)		
8-10 thousand	7 (15.6%)	5 (14.3%)	2 (4.4%)	1 (3.0%)		
10-20 thousand	17 (37.8%)	11 (31.4%)	13 (28.9%)	9 (27.3%)		
20-50 thousand	9 (20.0%)	7 (20.0%)	13 (28.9%)	8 (24.2%)		
>50 thousand	6 (13.3%)	6 (17.1%)	8 (17.8%)	8 (24.2%)		

Note. # Baseline characteristics of participants between groups were compared. IG: intervention group; WLG: waitlist group; PDD-NOS: pervasive developmental disorder not otherwise specified; CARS: Childhood Autism Rating Scale; M: mean; SD: standard deviation; [a] t test; [b] Chi-square test; [c] Fisher's exact test.

Table S6 Outcome measures of participants at baseline and exit in each group

Categories	IG			t_1 #	WLG			t_2 #
	Baseline(n=45)	Exit (n=35)	Lost (n=10)		Baseline (n=45)	Exit (n=33)	Lost(n=12)	
**SRS**								
Social awareness	11.42±2.90	11.54±3.09	11±2.21	0.517	11.96±2.7	11.85±2.60	12.25±3.05	–0.438
Social cognition	18.24±4.42	18.11±4.05	18.7±5.76	–0.366	18.11±4.94	17.39±4.59	20.08±5.52	–1.647
Social	32.64±8.57	33.11±8.35	31±9.57	0.684	33.44±8.6	33.03±8.65	34.58±8.74	–0.531
Social motivation	14.58±4.94	15.17±4.88	12.5±4.81	1.530	15.44±5.03	14.94±4.94	16.83±5.24	–1.120
Autistic	14.02±6.91	14.49±7.03	12.4±6.57	0.839	13.82±7.03	12.91±6.80	16.33±7.32	–1.464
Total scores	90.91±12.20	92.43±22.85	85.6±24.86	0.818	92.78±24.77	90.12±23.63	100.08±27.4	–1.199
**ATEC**								
Language	12.96±7.67	13.06±8.12	12.6±6.2	0.164	12.9±7.07	13.23±6.70	12.08±6.73	0.474
Sociability	17.78±7.51	18.03±7.68	16.9±7.2	0.415	18.83±7.89	19.41±6.73	17.17±9.22	0.867
Total scores	30.73±12.37	31.09±12.37	29.5±12.3	0.358	31.73±12.13	32.64±11.18	29.25±12.2	0.842
**PSI-SF**								
PD	34.91±8.91	35.77±8.18	31.9±11.06	1.219	34.8±9.74	34.64±11.02	35.25±5.1	–0.185
PCDI	30.47±8.61	31.49±9.10	26.9±5.63	1.506	29.71±6.59	28.85±6.55	32.08±6.37	–1.475

DC	35.78±8.61	35.80±8.54	35.7±9.3	0.032	36.51±7.22	35.91±7.48	38.17±6.44	–0.927
Total scores	101.16±22.59	103.06±22.21	94.5±23.86	1.058	101.02±17.89	99.39±19.34	105.5±12.72	–1.013
**PSOC**								
Satisfaction	31.96±6.47	31.00±6.05	35.3±7.09	–1.909	31.91±7.42	32.73±8.04	29.67±5.02	1.230
Efficacy	27.84±5.82	27.66±6.30	28.5±3.84	–0.400	28.91±5.72	29.39±5.76	27.58±5.62	0.938
Total scores	59.80±9.42	58.66±9.78	63.8±7.08	–1.546	60.82±8.02	62.12±7.47	57.25±8.72	1.851

Note. SRS: Social Responsiveness Scale; ATEC: Autism Treatment Evaluation Checklist; PSI-SF: Parenting Stress Index-Short Form; PD: parental distress; PCDI: parent-child dysfunctional interaction; DC: difficult child; PSOC: Parental Self-efficacy of Competence Scale; Characteristics were compared for Exit and Lost participants in intervention group and waitlist group, respectively. # All *P* values were above 0.05.
